# Anti-Biofilm Action of Cineole and *Hypericum perforatum* to Combat Pneumonia-Causing Drug-Resistant *P. aeruginosa*

**DOI:** 10.3390/antibiotics13080689

**Published:** 2024-07-24

**Authors:** Sourav Chakraborty, Piyush Baindara, Pralay Sharma, Austin Jose T, Kumaravel V, Raja Manoharan, Santi M. Mandal

**Affiliations:** 1 Department of Bioscience and Biotechnology, Indian Institute of Technology, Kharagpur 721302, India; souravchakraborty68323@gmail.com; 2 Animal Sciences Research Center, Division of Animal Sciences, University of Missouri, Columbia, MO 65211, USA; pbaindara@missouri.edu; 3 National Institute of Homoeopathy, Block-GE, Sector-III, Salt Lake, Kolkata 700106, India; pralay.nih@gov.in (P.S.); austinjose.nih@gov.in (A.J.T.); kumaravel.nih@gov.in (K.V.); 4 Department of Chemistry and Biochemistry, University of California San Diego, 9500 Gilman Dr, La Jolla, CA 92093, USA

**Keywords:** cineole, *Hypericum perforatum*, *Pseudomonas aeruginosa*, pneumonia, antibacterial agents, homeopathy

## Abstract

Hospital-acquired antibiotic-resistant pneumonia is one of the major causes of mortality around the world that pose a catastrophic threat. *Pseudomonas aeruginosa* is one of the most significant opportunistic pathogens responsible for hospital-acquired pneumonia and gained resistance to the majority of conventional antibiotics. There is an urgent need for antibiotic alternatives to control drug-resistant pneumonia and other related respiratory infections. In the present study, we explored the antibacterial potential of cineole in combination with homeopathic medicines against biofilm-forming drug-resistant *P. aeruginosa*. Out of 26 selected and screened homeopathic medicines, *Hypericum Perforatum* (HyPer) was found to eradicate biofilm-forming drug-resistant *P. aeruginosa* most effectively when used in combination with cineole. Interestingly, the synergistic action of HyPer and cineole was also found to be similarly effective against planktonic cells of *P. aeruginosa*. Further, the potential synergistic killing mechanisms of cineole and HyPer were determined by analyzing zeta membrane potential, outer membrane permeability, and DNA release from *P. aeruginosa* cells upon treatment with cineole and HyPer. Additionally, molecular docking analysis revealed strong binding affinities of hypericin (an active ingredient of HyPer) with the PqsA (a quorum sensing protein) of *P. aeruginosa*. Overall, our findings revealed the potential synergistic action of cineole and HyPer against biofilm-forming drug-resistant *P. aeruginosa*. Cineole and HyPer could be used in combination with other bronchodilators as inhalers to control the biofilm-forming drug-resistant *P. aeruginosa*.

## 1. Introduction

The rapid evolution of microbial drug resistance in clinical settings is the major cause of prolonged infection. Especially in the case of respiratory infections, pneumonia-causing bacteria such as *Streptococcus pneumoniae* and *P. aeruginosa* have been reported to develop resistance to multiple antibiotics [1,2]. The rapid emergence of drug resistance compromises treatment efficacy and subsequently results in a prolonged infection that ultimately progresses into severe respiratory distress [3]. Additionally, biofilm-forming bacteria such as *P. aeruginosa* are highly prone to develop resistance and significantly increase treatment time due to the complex dimensional structure of biofilms that creates biofilm-mediated drug resistance [4]. Overall, the rapid emergence of antibiotic-resistant bacteria generates huge pressure and an urgent requirement for new therapies and strategies to combat the prolonged bacterial infections caused by drug-resistant bacteria. The synergistic application or repurposing of known antibacterial compounds is one of the strategies to control the rapid evolution of drug resistance [5,6]. Further, natural medicines are another potential option to explore against drug-resistant pathogens, although detailed studies are required for the characterization of their respective bioactive constituents and their mechanisms of action. Especially, phytochemicals are one example of natural medicines and an enormous source of potential antibacterial agents; however, many of them have already been explored and are known for their potential applications [7,8]. Many homeopathic medicines are also composed of various phytochemicals only and have been proven to be potential remedies to fight against respiratory infections [9,10,11]. On the other hand, eucalyptus oil is one of the potential sources of phytochemicals including cineole that have demonstrated antibacterial effects against various microorganisms. Cineole is reported to exert its antibacterial effects by increasing bacterial surface charge and outer membrane permeability, the induction of lipid peroxidation via the generation of reactive oxygen species, and the loss of intracellular material including proteins and nucleic acid [12].

In the present study, 26 homeopathic medicines were selected based on their practical use in treating upper respiratory tract infections (URTIs), pneumonia, and chronic asthma within the context of Indian homeopathic practices. We screened the synergistic antibacterial action of cineole, and selected homeopathic medicines, against a hospital-acquired biofilm-forming drug-resistant strain of *P. aeruginosa*.

## 2. Results

### 2.1. Purification of Cineole and Selection of Homeopathic Medicines

In the present study, we performed the evaluation of selected homeopathic medicines in combination with cineole to combat a hospital-acquired drug-resistant strain of *P. aeruginosa*. We selected 26 commonly available homeopathic medicines that are usually prescribed for respiratory infections or illnesses (Table S1). On the other hand, cineole is known for its antibacterial properties against several pathogenic bacteria [13]. We freshly extracted and purified the eucalyptus oil using TLC to obtain cineole. The TLC-separated cineole was subsequently purified and collected on HPLC and quantified using the cineole standard, as described above, for further use (Figure S1). Next, to reconfirm the purity, a GC-MS analysis was performed. A total of four major oil components were identified in the sample, accounting for 96.9% of the whole composition. The purified oil sample was dominated by four compounds: 1,8-cineole (43.2%), α-pinene (24.9%), p-cymene (18.5%), and limonene (8.1%). The GC spectrum and corresponding mass spectrum of the sample, eluted at a retention time from 8.71 to 8.723 min, confirmed 1,8-cineole (m/z 154) as the major constituent of purified eucalyptus oil (Figure S2).

### 2.2. Screening of Homeopathic Medicines against Biofilm-Forming Drug-Resistant P. aeruginosa in Combination with Cineole

**Figure 1:**
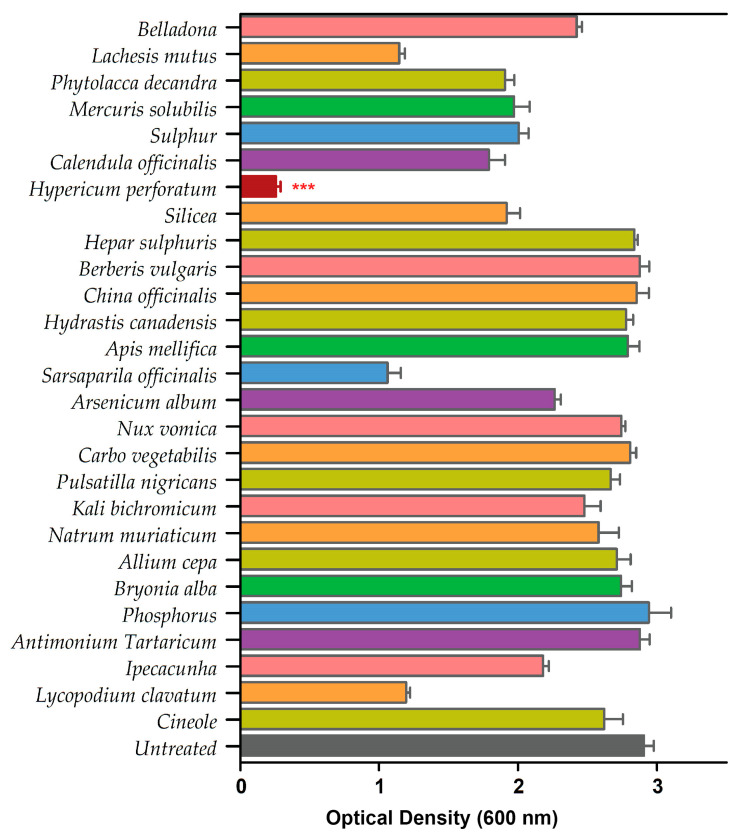
Primary screening of 26 homeopathic medicines against drug-resistant *P. aeruginosa*. *Hypericum perforatum* displayed the highest anti-biofilm activity, represented in a maroon color bar. Error bars show a standard deviation (SD) while statistical significance is considered at the level of *p* < 0.05 (indicated as red stars above bar). The experiment was performed three times independently in triplicate.

We performed a standard biofilm assay and screened all 26 homeopathic medicines in combination with cineole against *P. aeruginosa* biofilms (Table S1). The biofilm eradication assay revealed HyPer as the most effective homeopathic medicine when used in combination with cineole. The results of the biofilm eradication assay are represented as a heat map that clearly shows the potent and synergistic anti-biofilm action of HyPer and cineole (Figure 1). Interestingly, cineole alone was not found to be effective against drug-resistant *P. aeruginosa* biofilms at a high concentration of 125 µg/mL.

### 2.3. Hypericum Perforatum Showed Efficient Activity against Both Biofilm and Planktonic Cells of Drug-Resistant P. aeruginosa

**Figure 2:**
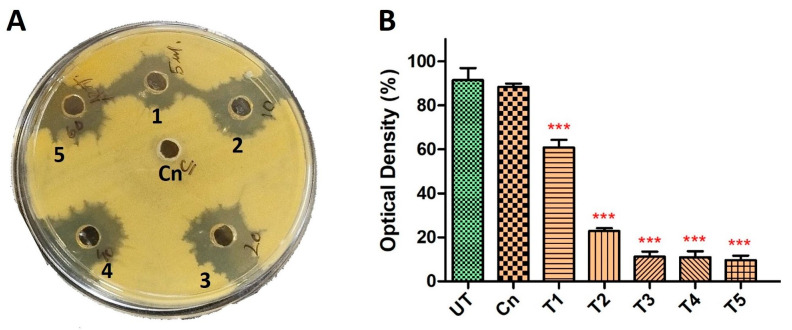
Synergistic antibacterial activity of HyPer and cineole. (**A**) Well diffusion assay displaying the synergistic action of HyPer and cineole. Well numbers 1, 2, 3, 4, and 5 indicate 5 μL, 10 μL, 20 μL, 40 μL, and 60 μL of HyPer (30 CH), respectively, in combination with 125 μg/mL of Cn. Cn alone was used as a control. (**B**) MIC determination of HyPer in combination with 125 μg/mL of cineole. T1, T2, T3, T4, and T5 indicate 5 μL, 10 μL, 20 μL, 40 μL, and 60 μL of HyPer (30 CH), respectively, in combination with 125 μg/mL of Cn. *P. aeruginosa* treated with Cn alone and 30% ethanol in PBS (UT) were used as controls. Error bars show a standard deviation (SD) while statistical significance is considered at the level of *p* < 0.05 (indicated as red stars above bars). All experiments were performed three times independently in triplicate.

To further explore the synergistic potential of HyPer and Cineole against *P. aeruginosa* biofilm, we determined the MIC values using different dilutions of HyPer in combination with 125 µg/mL of cineole. Our results suggested about 90% of *P. aeruginosa* biofilm eradication when 20 µL of HyPer was used with 125 µg/mL of cineole (T3) that remained static at higher treatments (T4 and T5) (Figure 2B). On the other hand, we also checked the synergistic action of HyPer and cineole against planktonic cells of *P. aeruginosa* employing a simple well diffusion assay. Interestingly, results showed a similar efficacy of HyPer and cineole combination against planktonic cells of *P. aeruginosa* where 20 µL of HyPer with 125 µg/mL of cineole showed the highest killing as revealed by the zone of inhibition (3) (Figure 2A). Interestingly, cineole alone was not found to be effective either against planktonic cells or biofilms of *P. aeruginosa* (Figure 2A,B).

### 2.4. Synergistic Killing Mechanism of Hypericum Perforatum with Cineole against P. aeruginosa

#### 2.4.1. Disruption of Membrane Zeta Potential

**Figure 3:**
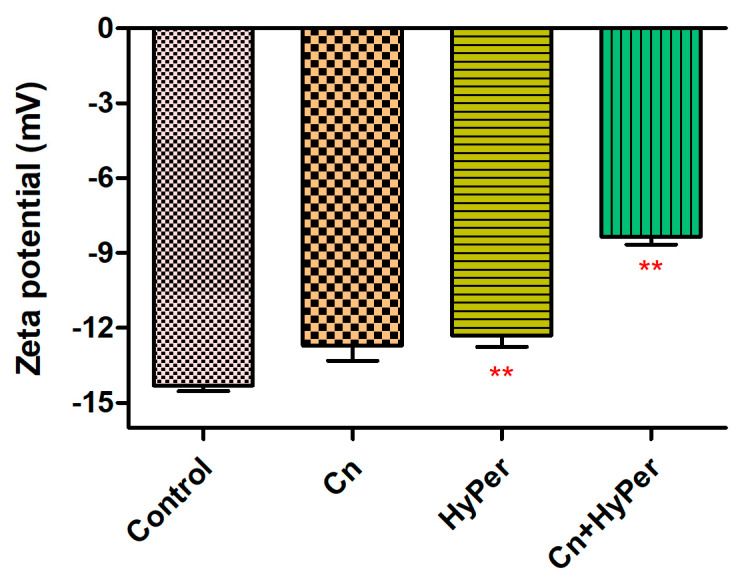
Membrane zeta potential of untreated and treated *P. aeruginosa* cells with cineole, HyPer, and a combination of both. *P. aeruginosa* treated with 30% ethanol in PBS was used as a control. Error bars show a standard deviation (SD) while statistical significance is considered at the level of *p* < 0.05 (indicated as red stars above bars). The experiment was performed three times independently in triplicate.

We confirmed the synergistic killing action of HyPer and cineole against both planktonic cells and biofilms of a hospital-acquired drug-resistant strain of *P. aeruginosa*. Further, to determine the molecular mechanism of killing, we performed the assay and measured the zeta potential of untreated bacterial cells in comparison to the treated bacterial cell with cineole, HyPer, and a combination of both. Due to the presence of LPS, Gram-negative bacterial cell membranes possess high negative charges that should be observed as increased values of zeta potential if the outer membrane is compromised or disrupted [14]. Our results demonstrated about a 40% increase in zeta membrane potential values upon treatment with HyPer in combination with cineole while only a slight increase was observed with treatment of HyPer and cineole alone (Figure 3). The results suggested the synergistic action of HyPer with cineole via the disruption of the zeta membrane potential of *P. aeruginosa* cells.

#### 2.4.2. Disruption of Outer Membrane Permeability

**Figure 4:**
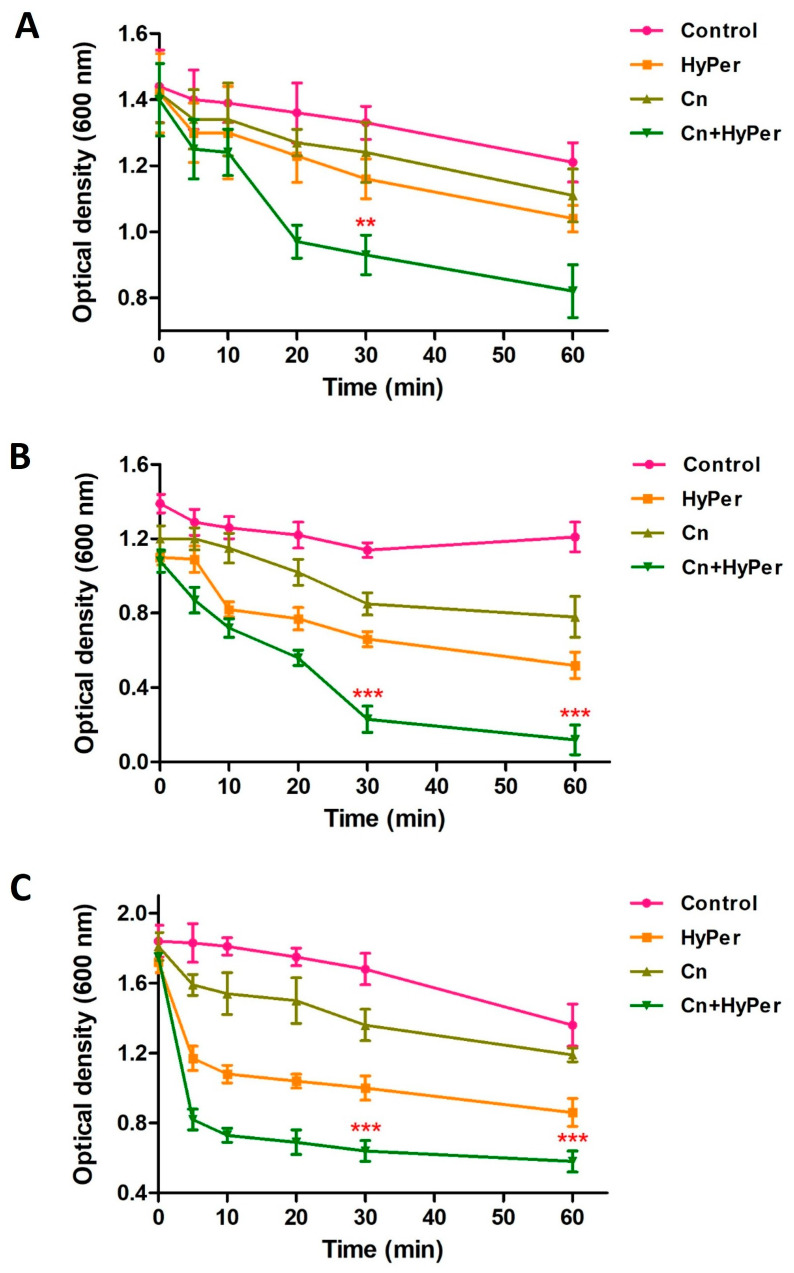
Outer membrane permeability of untreated and treated *P. aeruginosa* cells with cineole, HyPer, and a combination of both for different time points of treatment: (**A**) 1 h, (**B**) 2 h, and (**C**) 3 h. *P. aeruginosa* cells treated with 30% ethanol in PBS were used as a control. Error bars show a standard deviation (SD) while statistical significance is considered at the level of *p* < 0.05 (indicated as red stars). All experiments were performed three times independently in triplicate.

As the zeta potential assay revealed a significant increase upon treatment, we performed an outer membrane potential assay to confirm the outer membrane permeability and membrane-specific synergistic killing mechanism of HyPer and cineole. Normally, the outer membrane of Gram-negative bacteria is negatively charged and repels the other negatively charged molecules such as SDS. The compromised outer membrane of bacteria should result in a higher influx of SDS in terms of decreased absorbance at 600 nm. We performed an outer membrane permeability assay using low concentrations of SDS as a membrane permeabilization probe for the treatment time points of 1 h, 2 h, and 3 h (Figure 4). Our results revealed a time-dependent disruption of outer membrane permeability upon treatment with HyPer, cineole, and a combination of both. It is very clear that the increasing treatment time results in enhanced membrane permeabilization, which is also in agreement with the earlier experiments. Also, at all three different time points, treatment using the combination of HyPer and cineole showed the highest membrane permeabilization, which confirmed the synergistic membrane-specific killing mechanism of HyPer and cineole (Figure 4A–C).

#### 2.4.3. DNA Leakage

**Figure 5:**
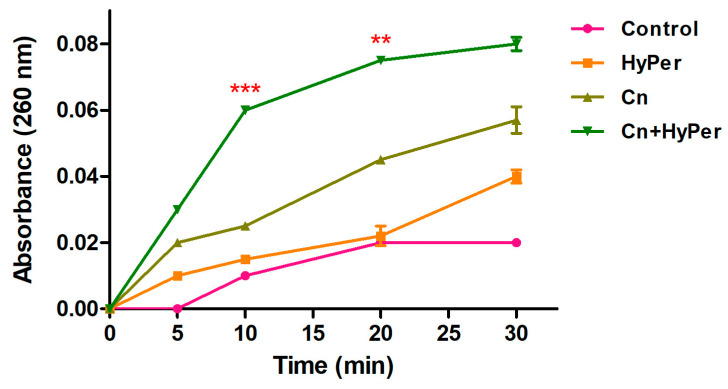
Intracellular leakage of untreated and treated *P. aeruginosa* cells with cineole, HyPer, and a combination of both for the time points of 0, 5, 10, 20, and 30 min. *P. aeruginosa* cells treated with 30% ethanol in PBS were used as a control. Error bars show a standard deviation (SD) while statistical significance is considered at the level of *p* < 0.05 (indicated as red stars). The experiment was performed three times independently in triplicate.

We performed a DNA leakage assay to further assure the membrane permeabilization upon treatment with HyPer in combination with cineole. Our DNA leakage assay was in agreement with the zeta potential and the outer membrane permeabilization assay results (Figure 5). The HyPer and cineole combined treatment showed the highest absorbance at 260 nm, which again confirmed the synergistic killing mechanism of HyPer and cineole.

### 2.5. Molecular Docking Revealed a Strong Interaction of Hypericin with PqsA of P. aeruginosa

**Figure 6:**
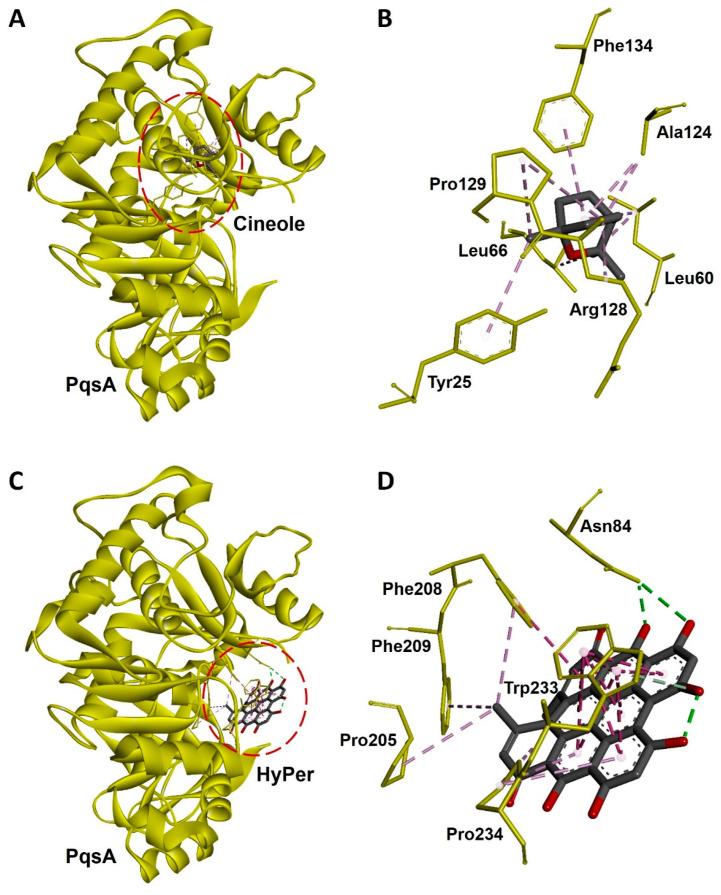
Molecular docking and interaction of cineole and hypericin with PqsA, a biofilm-forming protein of *P. aeruginosa*. (**A**) Docked complex of cineole and PqsA. The dotted red line circle highlights the position of cineole in the complex. (**B**) Three-dimensional representation of interacting amino acid residues of PqsA with cineole upon docking. (**C**) Docked complex of hypericin and PqsA. The dotted red line circle highlights the position of hypericin in the complex. (**D**) Three-dimensional representation of interacting amino acid residues of PqsA with hypercin upon docking. PqsA is shown as a yellow ribbon while cineole, hypercin, and interactive amino acid residues are shown as sticks. Interacting bonds are represented as dotted green, purple, and violet lines.

Hypericin is the active ingredient of HyPer which is known for its antibacterial action [15]. We performed the molecular docking experiment to check the molecular interaction of hypericin and cineole with the PqsA of *P. aeruginosa*. PqsA is a known quorum-sensing protein that plays an important role and promotes cell-to-cell communication during biofilm formation in *P. aeruginosa* [16]. Our molecular docking analysis revealed that hypericin binds almost 2.5 times more efficiently with PqsA of *P. aeruginosa* when compared to cineole (Figure 6A,C). The docking scores were −70.21 and −176.99 Kcal/mol for cineole/PqsA and hypericin/PqsA complex, respectively (Table 1). We further explored the interacting amino acid residues involved in the binding of cineole and hypericin with PqsA (Figure 6B,D). Overall, hypericin’s interaction with PqsA revealed a compact pocket-like structure that resulted in strong binding affinities (Figure 6D). Possibly, the formation of this compact pocket-like structure between hypericin and PqsA results in a strong binding that subsequently leads to the efficient destruction of the biofilm network in *P. aeruginosa*.

## 3. Discussion

Pneumonia is a chronic inflammation of the alveoli of the lungs caused by infections with different kinds of bacteria, viruses, and fungi. Specifically, biofilm-forming bacteria such as *S. pneumoniae* and *P. aeruginosa* are commonly found in both acute and chronic lung infections in humans [17,18]. Additionally, biofilm-forming bacteria are more resistant to eradication due to complex 3D biofilm structures and so more prone to develop drug resistance [19,20,21]. Biofilm-forming bacteria are one of the major players in the rapid evolution of drug resistance that needs serious attention for the development of new strategies or new formulations to combat it. Quorum sensing plays an important role during biofilm formation, and thus quorum sensing proteins of *P. aeruginosa* such as pqsA could be a potential target to eradicate drug-resistant biofilms. pqsA is a cell-signaling protein that leads to the synthesis of the signaling molecule PQS. PQS functions as a linker molecule between las and rhl quorum sensing modules during biofilm formation. So, the inhibition of pqsA results in PQS disruption and thus biofilm degradation. Additionally, the drug repurposing or synergistic action of available drugs is one of the strategies to eradicate or combat biofilm-forming pathogenic bacteria and disease. Homeopathic medicines or phytochemicals are one of the potential therapeutic agents that can be used or explored to fight against biofilm-forming drug-resistant bacteria in respiratory infections [9,22]. Homeopathic therapy has also been found to be effective in the recent past during the COVID-19 pandemic, which further suggested to explore the potential of homeopathic medicines against drug-resistant bacteria causing lung infections [23,24].

In the present study, we explored the antibacterial potential of selected homeopathic medicines in combination with cineole against a hospital-acquired biofilm-forming drug-resistant strain of *P. aeruginosa*. Cineole is already known for its antibacterial activity; however, in the present study, we aimed to check its synergistic potential with selected homeopathic medicines against biofilm-forming drug-resistant *P. aeruginosa* (Table 1). We freshly extracted and purified cineole from eucalyptus leaves; however, cineole was not found to be effective against the planktonic cells and biofilms of *P. aeruginosa* (Figure 2). Interestingly, when cineole was used in combination, it was found to improve the antibacterial effect of homeopathic medicines (Figure 1). Out of all 26 homeopathic medicines, HyPer was found to be most effective in eradicating biofilms. We further determined the antibacterial action of HyPer against both planktonic cells and biofilms of *P. aeruginosa* using a well assay and a biofilm eradication assay, respectively. Interestingly, HyPer was found to be equally efficient against planktonic cells and biofilms of *P. aeruginosa* (Figure 2). Next, the membrane-specific synergistic killing mechanism of HyPer and cineole was determined and expressed in terms of zeta membrane potential, outer membrane potential, and DNA release upon treatment, which were in agreement with the biofilm eradication assay (Figure 3, Figure 4 and Figure 5). Finally, molecular docking analysis revealed a strong interaction of HyPer’s active compound hypericin with the PqsA of *P. aeruginosa*, suggesting the possible reason behind the efficient biofilm eradication by the synergistic action of HyPer and cineole (Figure 6). Overall, the results suggested the efficient synergistic killing of drug-resistant *P. aeruginosa* using the combination of HyPer and cineole that can be further explored for therapeutic applications.

## 4. Conclusions

The present study suggested the combinatorial use of HyPer and cineole that can be used to fight against biofilm-forming drug-resistant *P. aeruginosa*. By demonstrating the efficient antibacterial activity against both planktonic cells and biofilms of *P. aeruginosa*, the therapeutic application of HyPer and cineole could be a new approach to combat the drug resistance of *P. aeruginosa*. However, potential immune responses of this combinatorial therapy remain elusive, along with other adverse effects, if any, which were not investigated in the present study. Also, further studies are required to check the efficacy of HyPer and cineole in in vivo systems. Further, molecular interactions of hypericin with PqsA were determined using in silico tools only, which warrants further in vitro and in vivo studies to determine the synergistic killing mechanism of HyPer and cineole. Overall, despite the facts and limitations, the finding of the current study provides clues for future studies regarding the synergistic potential of HyPer and cineole to combat drug-resistant *P. aeruginosa* that gives new hope for the development of novel combinatorial therapies as alternatives to traditional antibiotics to tackle the rapid evolution of drug-resistance and biofilm-forming pathogenic bacteria.

## 5. Material and Methods

### 5.1. Plant Materials

Fresh and young eucalyptus leaves were collected, washed, and used to obtain eucalyptus oil. The oil was obtained through a process of steam distillation, which extracts the volatile compounds responsible for its characteristic aroma and therapeutic properties. In total, 500 g of freshly dried leaves were coarsely crushed and boiled with 1 L of distilled water for about half an hour in a Clevenger-type apparatus. The water vapors generated in the heated flask traveled through the crushed and boiled leaves, picked up essential oil, and condensed in the condenser. Subsequently, the extract was decanted and collected after condensation. Next, essential oil was separated by centrifugation at 12,000× *g* for 10 min. The final yield of essential oil was approximately 0.84% (*w*/*w*) [25].

### 5.2. Homeopathic Medicines and Cineole

We selected homeopathic medicines based on recommendations according to the symptoms mentioned in the literature (https://www.drhomeo.com/homeopathic-treatment/homeopathic-treatment-pneumonia/) (https://www.medicalnewstoday.com/articles/325376#research), accessed on 15 August 2023. We specifically focused on the homeopathic medicines used to treat pneumonia. Based on the selection, we purchased 26 homeopathic medicines (30 CH potency) from a GMP-certified company. The homeopathic medicines used in the current study are as follows: *Belladonna*, *Lachesis mutus*, *Phytolacca decandra*, *Mercurius solubilis*, *Sulphur*, *Calendula officinalis*, *Hypericum perforatum*, *Silicea*, *Hepar sulphuris*, *Berberis vulgaris*, *China officinalis*, *Hydrastis canadensis*, *Apis mellifica*, *Sarsaparilla officinalis*, *Arsenicum album*, *Nux vomica*, *Carbo vegetabilis*, *Pulsatilla nigricans*, *Kali bichromicum*, *Natrum muriaticum*, *Allium cepa*, *Bryonia alba*, *Phosphorus*, *Antimonium tartaricum*, *Ipecacuanha*, and *Lycopodium clavatum* (Table S1). All homeopathic medicines had 30% ethanol (*v*/*v*) content. 1, 8-Cineole standard with 99% purity was purchased from Sigma-Aldrich, MA, USA.

### 5.3. Bacterial Strain and Media

*P. aeruginosa* is one of the major pathogens causing hospital-acquired pneumonia. To check and compare the efficacy of cineole and homeopathic medicines, a clinically isolated drug-resistant strain of *P. aeruginosa* was obtained from the Department of Microbiology, Bankura Sammilani Medical College and Hospital, Kenduadihi, Bankura 722102, West Bengal, India. *P. aeruginosa* was grown on nutrient agar (NA) and nutrient broth (NB) for single colonies and liquid cultures, respectively. NA and NB were purchased from Hi Media, India.

### 5.4. Thin Layer Chromatography

A small silica-coated aluminum plate was used to perform thin-layer chromatography (TLC) to separate the phytochemical constituents of eucalyptus oil. A single drop of eucalyptus oil was spotted on the silica plate and allowed to dry before running TLC. A combination of hexane–chloroform (6:4) was used as a mobile phase to run TLC in a closed chamber [26]. After running TLC, when the mobile phase solvent reached the top of the silica plate, the plate was allowed to be air-dried, and subsequently visualized under UV light and also in an iodine-saturated chamber to visualize the separated components of eucalyptus oil [27].

### 5.5. High-Performance Liquid Chromatography

Further purification of eucalyptus oil was performed using high-performance liquid chromatography (HPLC), as described previously [28,29]. Hydro-distilled eucalyptus oil was diluted in n-hexane and subsequently analyzed using a ZORBAX-Eclipse XDB-C18 column (4.6 × 150 mm, particle size 5 µm) by HPLC on a 1260 Infinity instrument (Agilent Technologies, Santa Clara, CA, USA). HPLC-grade water containing 0.1% trifluoroacetic acid (TFA) was used as solvent A while 0.1% acetonitrile was used as solvent B and monitored via a UV detector at 220 nm. A gradient of solvent B was applied as 0–60% for the first 5 min, 60–80% for the next 12 min, and 80–100% for the last 20 min at a flow rate of 1 mL/min for the better separation of the sample. The samples were filtered using 0.22 µ Millex-SR filters (Millipore, Billerica, MA, USA) before injection in the HPLC system. After collection, each fraction was collected and concentrated using a vacuum evaporator (Eppendorf, CT, USA). Purified cineole was quantified using the 1, 8-Cineole standard obtained from Sigma-Aldrich, Waltham, MA, USA.

### 5.6. Gas Chromatography–Mass Spectrometry Analysis

An Agilent 6890N gas chromatograph equipped with 5973N mass spectrometer was utilized to ascertain the chemical composition of eucalyptus oil. A 5% phenylmethylpolysiloxane HP-5MS (30 m × 0.25 mm i.d. × 0.1 µm film thickness, Agilent, Folsom, CA, USA) and a polyethylene glycol DB-WAX (30 m × 0.25 mm i.d. × 0.25 µm film thickness, Agilent) were used as stationary phases for the analysis. The operational parameters (the heat of the oven, the split ratio, the temperature of the injector and detector, etc.) were the same as those documented by Pavela et al., 2019 [30].

### 5.7. Agar Well Diffusion Assay

Purified cineole and HyPer were checked alone and in combination to check the activity against *P. aeruginosa* using an agar well diffusion assay on NA plates. The target bacterial strain *P. aeruginosa* was grown for 2–4 h (or up to 0.5 OD) by inoculating a single colony from the NA plate in 15 mL of glass tubes containing 5 mL of NB. Test tubes containing *P. aeruginosa* cultures were then preserved at 4 °C for further use. NA plates containing *P. aeruginosa* were prepared by spreading 15 µL of test strain on the plate using a sterile glass spreader. Subsequently, wells were punched on the plates using a sterile cork borer. Different dilutions of HyPer with cineole were prepared in PBS while purified cineole dilutions were prepared using ethanol. All the test dilutions of HyPer with cineole were adjusted to the final volume of 100 µL and were loaded to the respective wells on the NA plate. Plates loaded with the samples were further incubated overnight in a 33 °C incubator to check the antimicrobial activity of test dilutions. Cineole alone at a concentration of 125 µg/mL was used as a control. If the target bacterial strain *P. aeruginosa* was inhibited by the used test dilutions, it was observed as a zone of inhibition surrounding the punched wells.

### 5.8. MIC Determination

The MIC (minimum inhibitory concentration) of HyPer in combination with cineole was determined using a 96-well microtiter plate dilution assay [28,29]. Different dilutions of antibiotic HyPer with cineole were prepared in PBS to determine the MIC values against *P. aeruginosa*. To determine MIC, the test strain *P. aeruginosa* (~2 × 10^4^ cells) was treated with 5, 10, 20, 40, and 60 µL dilutions of HyPer in combination with 125 µg/mL of cineole. OD was observed after 24 h of treatment. *P. aeruginosa* treated with 125 µg/mL cineole, and 30% ethanol in PBS were used as controls. The experiment was performed three times independently in triplicate and then analyzed for final results.

### 5.9. Biofilm Eradication Assay

To evaluate the anti-biofilm activity of cineole and HyPer, a biofilm eradication assay was performed using pre-formed biofilms, as described earlier [31]. In a 96-well microtiter plate, 200 μL of *P. aeruginosa* culture with 0.01 OD was added in each well and incubated for 48 h at 37 °C in static condition for the biofilm formation. Following the incubation, unbound and planktonic cells were removed and, subsequently, the adhered biofilms in the bottom of the 96-well plates were washed with 100 μL of PBS three times. Test dilutions of all 26 homeopathic medicines (100 µL each) in combination with cineole (125 µg/mL) were prepared in PBS, and the final volume was made to 200 μL before adding to the biofilm-containing wells. PBS was used as a control. The test microtiter plate was again incubated overnight under static conditions at 37 °C to check the biofilm eradication potential of the test compounds. On the next day, unbound cells and media were aspirated, and the adhered biofilms were washed three times with PBS. Subsequently, biofilms were stained with 100 μL of 0.1% crystal violet (CV) solution (Sigma-Aldrich). After 20 min of incubation, CV was removed and biofilms were washed three times with PBS. The CV-stained biofilms were then resuspended in 70% ethanol with gentle mixing, and optical absorbance was recorded at 600 nm.

### 5.10. Microscopy

*P. aeruginosa* biofilms were formed in 96-well microtiter plates, as described above. The respective test dilutions of cineole, HyPer, and a combination of both were prepared in PBS, and the final volume was adjusted to 200 μL before adding to the biofilm-containing wells. Representative images of treated biofilms upon the respective treatments were captured under a light microscope (Zeiss, Dublin, CA, USA).

### 5.11. Determination of Zeta Potential

Malvern Zetasizer Nano ZS devices (Malvern Devices Ltd., Worcestershire, UK), equipped with a titration device MPT-2 (Malvern Instruments Ltd., Worcestershire, UK) and a 4 mV He-Ne laser illuminating at 633 nm, were used to perform dynamic light scattering (DLS) measurements to determine the zeta potential of *P. aeruginosa* after treatment with cineole, HyPer, and a combination of both. The devices were switched on at least 30 min before the measurements to guarantee laser stability [32]. The measurements were carried out using disposable polycarbonate folded capillary cells with gold-plated beryllium copper electrodes (Malvern DTS1061 or DTS1070). Before sample loading, the capillary cells were pre-treated with analytical-grade ethanol and then thoroughly washed with HPLC-grade water. Furthermore, before being filled, the capillary cells were washed with the sample to eliminate dilution effects from residual water.

### 5.12. Outer Membrane Permeability Assay

To explore the killing mechanism of cineole and HyPer against *P. aeruginosa*, an outer membrane permeabilization assay was performed using sodium dodecyl sulfate (SDS) [33,34]. Overnight-grown cultures of *P. aeruginosa* were centrifuged at 12,000× *g* for 10 min and washed with PBS twice. The washed bacterial cells were treated and incubated with cineole, HyPer, and a combination of both for 1, 2, and 3 h. After incubation for treatment points, each sample was washed twice with PBS to remove any traces of treatment compounds. Next, each test sample was treated with SDS at 0.1% (*w*/*v*) of the final concentration while the control samples were treated with PBS. SDS functions as a permeabilizing probe and can cause cell death upon sudden influx through the permeabilized outer membrane. Viable cell counts were determined and quantified in terms of OD (600 nm) at different time points of 0, 5, 10, 20, 30, and 60 min using a spectrophotometer.

### 5.13. DNA Leakage Assay

A DNA leakage assay was used to determine the killing potential and mechanism of cineole, HyPer, and a combination of both, as described earlier [35]. Overnight-grown cultures of *P. aeruginosa* were centrifuged at 12,000× *g* for 10 min and washed with PBS twice before treatment with the respective compounds. Different time intervals of 0, 5, 10, 20, and 30 min were used to determine the effect of treatment and, subsequently, DNA release was recorded in terms of OD at 260 nm using a spectrophotometer.

### 5.14. Protein Retrieval and Preparation

To reveal the biofilm-specific mechanism of action of cineole and HyPer against *P. aeruginosa*, the crystal structure of the N-terminal domain of PqsA from *P. aeruginosa* (PDB ID: 5OE3) was retrieved in PDB format from the RCSB PDB database (http://www.rcsb.org). PqsA is an autoinducer for cell-to-cell communication that plays an important role in *P. aeruginosa* biofilm formation. Further, the PqsA protein structure was refined by removing preoccupied ligands, ions, and water (H_2_O) molecules by using Chimera 1.15 (https://www.cgl.ucsf.edu/chimera). Active site residues of PqaA were predicted by using Fpocket (https://bioserv.rpbs.univ-paris-diderot.fr/services/fpocket, accessed on 15 August 2023) online tool [36]. Finally, the PqsA protein was modified again by using Chimera 1.15 to remove chains that were not involved in the active site [37].

### 5.15. Ligand Preparation

The 2D structures of cineole (CID: 2758) and hypericin (CID: 3663), an active compound of HyPer, were downloaded from PubChem (https://pubchem.ncbi.nlm.nih.gov) in SDF format. The SDF format structures were further prepared in 3D PDB format by using Chimera 1.15 (https://www.cgl.ucsf.edu/chimera) and visualized in Biovia Discovery studio [37].

### 5.16. Molecular Docking Analysis

Molecular docking experiments were performed using the HDOCK online server (http://hdock.phys.hust.edu.cn) [38]. The best protein–drug complex was selected from among the top ten conformers based on docking scores represented as free binding energy, kcal/mol. Next, the docked protein–drug complexes were selected for further analysis of non-bonded interactions. The interaction analysis was conducted using PyMOL and Biovia Discovery Studio visualization software packages and visualized as described earlier [37].

### 5.17. Statistical Analysis

All results and data represented in the present study are based on the mean ± standard deviation of the mean (SD). Column statistics for non-parametric data were analyzed using one-sample *t*-tests. The results for all the respective experiments were finalized and considered significant only when *p* < 0.05 in all experiments. All experiments were conducted three times independently in triplicate along with the respective controls.

## Figures and Tables

**Table 1:** Molecular docking scores of cineole and hypericin with PqsA of *P. aeruginosa*, represented as free binding energy (kcal/mol).

Free Binding Energy (kcal/mol)		
	Cineole	Hypericin
PqsA	−70.21	−176.99

## Data Availability

The original contributions presented in the study are included in the article/Supplementary Material, and further inquiries can be directed to the corresponding author/s.

## Supplementary Materials

The following supporting information can be downloaded at: https://www.mdpi.com/article/10.3390/antibiotics13080689/s1.

## Abbreviations

*w*/*w*: Weight by weight; g: Gravity; *v*/*v*: Volume by volume; mm: Millimeter; nm: Nanometer; µm: Micrometer; OD: Optical density; mL: Milliliter; µg: Microgram; h: Hours; LPS: Lipopolysaccharide.
