# Supplementary material for: Anti-Biofilm Action of Cineole and Hypericum perforatum to Combat Pneumonia-Causing Drug-Resistant P. aeruginosa
Source: Antibiotics (Basel). 2024 Jul 24;13(8):689. doi: 10.3390/antibiotics13080689 (PMC11350762; doi:10.3390/antibiotics13080689)

Anti-biofilm action of cineole and *Hypericum perforatum* to combat pneumonia causing drug resistant *P. aeruginosa*

Sourav Chakraborty[1#], Piyush Baindara[2#], Pralay Sharma[3], Austin Jose T[3], Kumaravel V[3], Raja Manoharan[3]*, Santi M. Mandal[1, 4]*

[1]Department of Bioscience and Biotechnology, Indian Institute of Technology Kharagpur 721302, India

[2]Animal Sciences Research Center, Division of Animal Sciences, University of Missouri, Columbia, MO 65211, USA

[3]National Institute of Homoeopathy, Block - GE, Sector - III, Salt Lake, Kolkata 700106, West Bengal, India

[4]Department of Chemistry and Biochemistry, University of California San Diego, 9500 Gilman Dr, La Jolla, CA 92093, USA

[#] These authors contributed equally.

***Correspondence**

Raja Manoharan, PhD
National Institute of Homoeopathy
Block - GE, Sector - III, Salt Lake, Kolkata - 700106, WB, India
E. mail: drrajanih@gmail.com

Santi M. Mandal, PhD
Department of Bioscience and Biotechnology
Indian Institute of Technology Kharagpur, 721302, India
E.mail: mandalsm@gmail.com

Table S1. Selected homeopathic medicines used in the present study and their active ingredients.

**Homeopathic medicine**	**Chemical constituents**
**Belladonna**	Atropine
	Hyoscyamine
	Scopolamine
**Lachesis**	Serine
	Metalloproteinases
	Phospholipases A2
	Bradykinin-potentiating peptides
**Phytolacca**	Triterpene saponins
	Tannin
	Resin pokeweed mitogen
**Mercurius**	Mercury nitrate
**Sulphur**	Finely powdered sulfur
**Calendula**	Mucilage
	Calendic acid
	Patulitrin
	Patuletin
	Triterpenoid saponins
	Triterpenoids
	Tetraterpenes/Carotenoids
**Hypericum perforatum**	Hypericin
	Pseudohypericin
	Amentoflavone
	Hyperforin
	Quercetin
	Hyperoside
	α-Pinene
	Pseudohypericin
	Adhyperforin
	Myrtenol
**Silica**	Sillicon oxide
**Hepar sulfuris**	Impure calcium carbonate
**Berberis**	Alkaloids Berberine
	Oxyberberine
	Berbamine
	Aromoline
	Protoberberine alkaloid karachine

	Palmatine
	Oxycanthine
	Taxilamine
	Tannins
	Flavoxanthin
	Auroxanthin
	Lutein
	Hydrastine
	Hydrastinine
	Canadine
**Chimaphila**	5-hydroxymethylfurfural
	Isohomoarbutin,
	Methyl salicylate
	Taraxerol
	beta-sitosterol
	Ursolic acid
	Nonacosane
	Hentriacontane
	Isohomoarbutin
	Renifolin
	Arbutin
	Avicularin
	Hyperoside
**Hydrastis**	Isoquinoline alkaloids
	Hydrastine
	Berberine
	Hydrastinine
	Canadine
**Apis**	Retinol
	Thiamin
	Riboflavin
	Tocopherol
	Antihemorrhagic vitamin
	Niacin
**Sarsaparilla**	Saponin glycoside
	Sarsasapogenin
	Dextrose
**Arsenicum album**	Arsenopyrite
	Cobalt

**Nux vomica**	Strychnine
	Brucine
**Carbo veg**	Carbon
**Pulsatilla**	Anemone
**Kali bichromicum**	Bichromate of potash
**Natrum muriaticum**	Common salt, sodium, and chloride
**Allium cepa**	Allicin
	Quercetin
	Fisetin
	Diallyl disulphide
	Diallyl trisulphide
**Bryonia**	Cucurbitacin glucosides
	Trihydroxyoctadecadienoic acids
**Phosphorus**	Phosphorus
**Antimonium Tart**	Tartar emetic
	Antimony-potassium tartrate
**Ipecac**	Alcohol extraction
	Plant alkaloids
	Cephaeline
	Methyl-cephaeline (emetine)
**Lycopodium**	Oxolycoclavinol
	Oxoserratenetriol
	Tohogenol
	Tohogeninol

Figure S1. HPLC chromatogram (blue line) of cineole purification using the thin layer chromatography purification of Eucalyptus oil. The pointed peak indicates the fraction containing cineole. The red dotted circle showed the separation of cineole on the TLC plate that subsequently ran on HPLC, resulting in a respective peak (indicated by a black arrow).

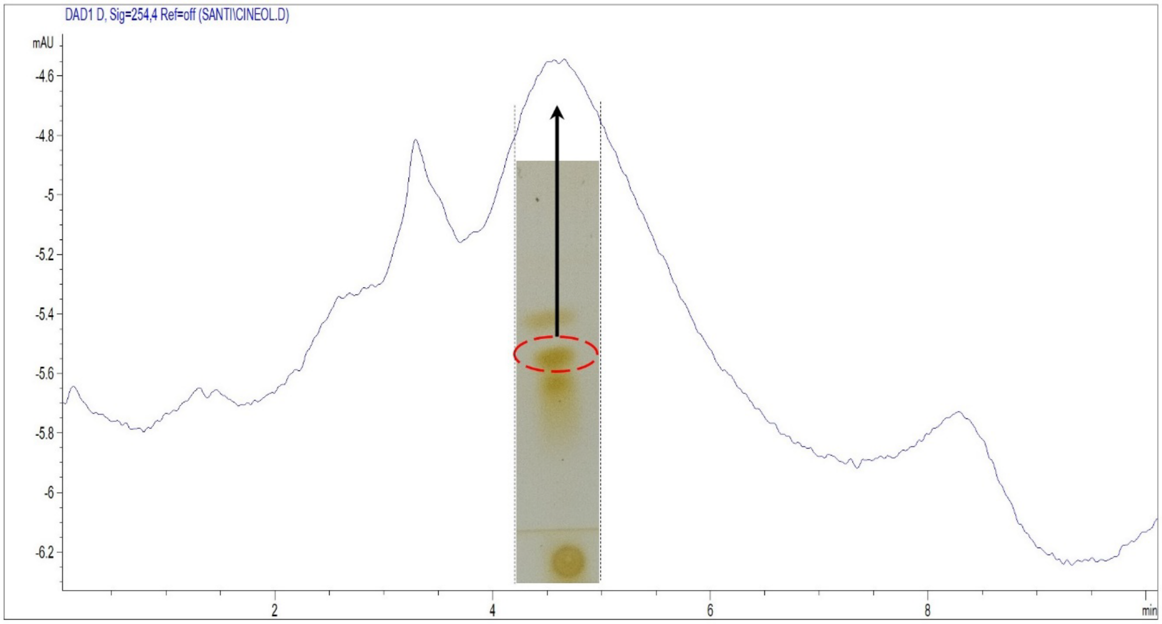

Figure S2. GC-MS analysis of extracted eucalyptus oil obtained after steam distillation from the young leaves of *Eucalyptus globulus*. Corresponding MS spectra from the GC spectrum (retention time 8.71 to 8.723) are shown in red outline (inset), confirming 1,8-cineole (m/z 154) as a major constituent of extracted eucalyptus oil.

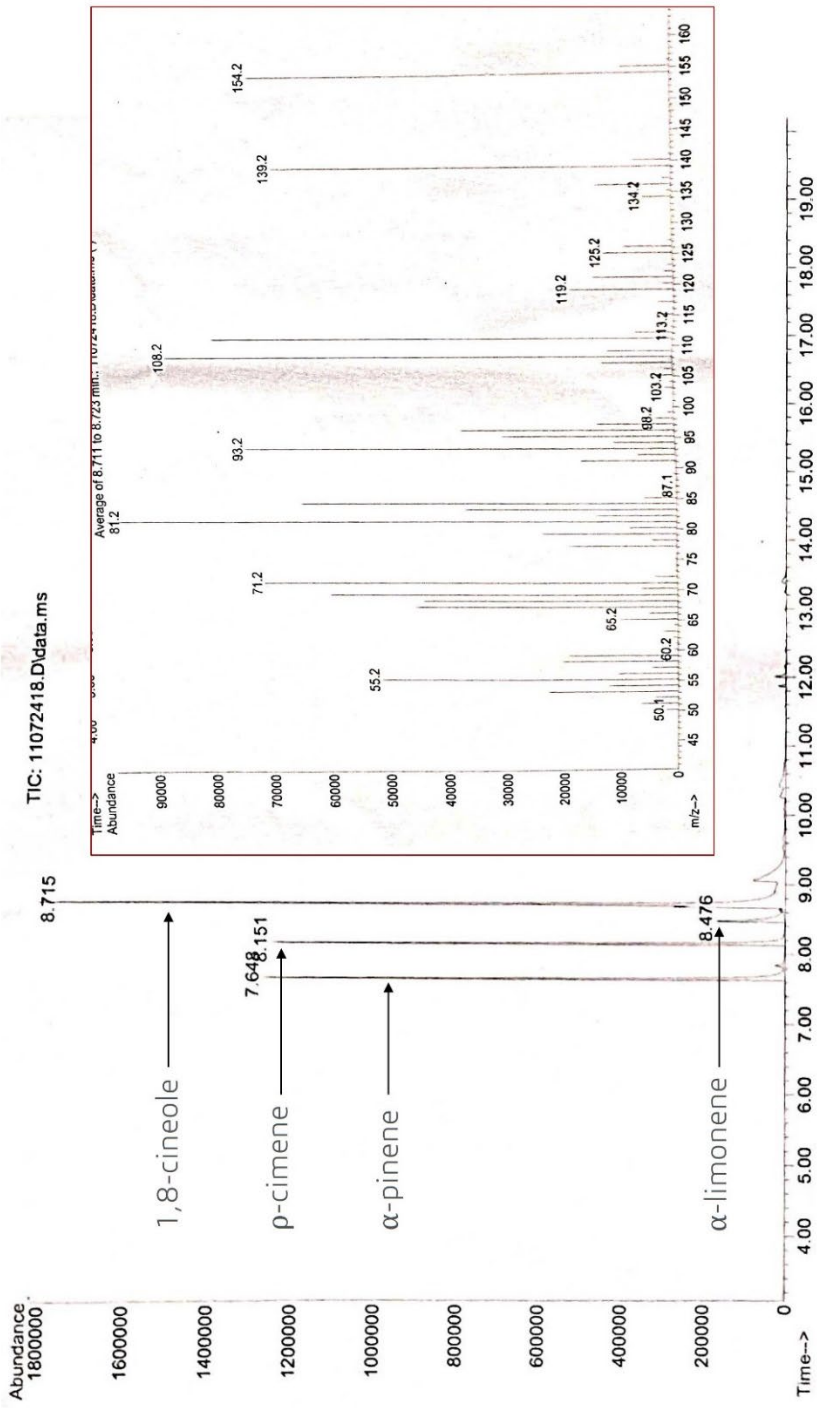

Supplement: Supplementary file 1 [file antibiotics-13-00689-s001.zip › antibiotics-3059332-supplementary.pdf]
